# Understanding intra- and interprofessional team and teamwork processes by exploring facility-based neonatal care in kenyan hospitals

**DOI:** 10.1186/s12913-022-08039-6

**Published:** 2022-05-13

**Authors:** Joyline Jepkosgei, Mike English, Mary B Adam, Jacinta Nzinga

**Affiliations:** 1 Health Services Unit, KEMRI-Wellcome Trust Research Programme, P. O. Box 43640 – 00100, 197 Lenana Place, Lenana Road, Nairobi, Kenya; 2 Centre for Tropical Medicine and Global Health, Nuffield Department of Medicine, University of Oxford, Oxford, UK; 3 AIC Kijabe Mission Hospital, Kijabe, Kenya; 4 Africa Consortium for Quality Improvement Research in Frontline Healthcare (ACQUIRE), Nairobi, Kenya

**Keywords:** Newborn care, Intra-professional, Interprofessional, Teamwork, Team processes

## Abstract

**Background:**

Within intensive care settings such as neonatal intensive care units, effective intra- and interprofessional teamwork has been linked to a significant reduction of errors and overall improvement in the quality of care. In Kenya, previous studies suggest that coordination of care among healthcare teams providing newborn care is poor. Initiatives aimed at improving intra- and interprofessional teamwork in healthcare settings largely draw on studies conducted in high-income countries, with those from resource-constrained low and middle countries, particularly in the context of newborn care lacking. In this study, we explored the nature of intra- and interprofessional teamwork among health care providers in newborn units (NBUs) of three hospitals in Kenya, and the professional and contextual dynamics that shaped their interactions.

**Methods:**

This exploratory qualitative study was conducted in three hospitals in Nairobi and Muranga Counties in Kenya. We adopted an ethnographic approach, utilizing both in-depth interviews (17) and non-participant observation of routine care provision in NBUs (250 observation hours). The study participants included: nurses, nursing students, doctors, and trainee doctors. All the data were thematically coded in NVIVO 12.

**Results:**

The nature of intra- and interprofessional teamwork among healthcare providers in the study newborn units is primarily shaped by broader contextual factors and varying institutional contexts. As a result, several team types emerged, loosely categorized as the ‘core’ team which involves providers physically present in the unit most times during the work shift; the emergency team and the temporary ad-hoc teams which involved the ‘core’ team, support staff students and mothers. The emergence of these team types influenced relationships among providers. Overall, institutionalized routines and rituals shaped team relations and overall functioning.

**Conclusions:**

Poor coordination and the sub-optimal nature of intra-and interprofessional teamwork in NBUs are attributed to broader contextual challenges that include low staff to patient ratios and institutionalized routines and rituals that influenced team norming, relationships, and team leadership. Therefore, mechanisms to improve coordination and collaboration among healthcare teams in these settings need to consider contextual dynamics including institutional cultures while also targeting improvement of team-level processes including leadership development and widening spaces for more interaction and better communication.

**Supplementary information:**

The online version contains supplementary material available at 10.1186/s12913-022-08039-6.

## Background

The quality of care provided to sick newborns in many low-and-middle-income countries (LMICs) hospitals is sub-optimal [1]. Newborn care requires multiple interventions delivered in complex contexts characterized by multiple actors including clinicians, nurses, nutritionists, and families, among others [2]. Coordination of care among these actors is critical; with quality care reliant on effective interdisciplinary teamwork [3, 4]. Nurses play a central role as coordinators of care and they provide information linkages between all healthcare actors in such inpatient Newborn Unit settings (NBUs) [5, 6]. However, there is a paucity of literature on how care is coordinated among interprofessional care delivery teams in NBUs, especially in LMICs. In this paper, we focus on how individuals within and between cadres interact, communicate and coordinate care provision in Kenyan NBUs.

Interprofessional teamwork is defined as “a dynamic process involving two or more healthcare professionals with complementary backgrounds and skills, sharing common health goals and exercising concerted physical and mental effort in assessing, planning, or evaluating patient care” [7]. Interprofessional teamwork is increasingly recognised as important due to the complex and dynamic nature of healthcare organisations, the growing specialization of healthcare professions and the increasing complexity of healthcare [8, 9]. Interprofessional teamwork is essential in improving coordination of care, reducing duplication of work, and enhancing patient safety and quality of care [4, 10]. Furthermore, it may lead to improved well-being and job satisfaction among employees while poor intra- and interprofessional teamwork is associated with higher rates of missed care, medical errors, and patient adverse events [11, 12].

Studies from high-come settings suggest that interprofessional teamwork in inpatient clinical settings is sub-optimal [13, 14], with interprofessional interactions often opportunistic, happening informally throughout different work shifts [15]. It is further undermined by tensions over role boundaries, contested professional hierarchies, tribalism, and professionalized power relationships [10, 15, 16]. Although positive working relationships and cooperation have been reported within and between interprofessional teams, these only happen in supportive work environments. These are environments characterized by a cooperative team, working without professional competition and including efficient means of communication such as having central case notes to avoid duplication [17, 18]. Contextual factors such as institutional cultures, tensions created by gaps in the rota, and workload have been shown to limit time for communication within teams [16].

Over the years, there has been a move towards a recognition of the influence of both the structural aspects referred to as ‘hardware elements’ of teams (which include team structure) [19] and the ‘software elements’ (e.g. team norms, relationships, communication and leadership processes) [18, 20, 21] on interprofessional team functioning and performance. As such, there have been a variety of interventions aimed at improving team effectiveness, such as simulation training and crew resource management training (CRM) which are focused on improving non-technical skills of teams including communication, leadership and team behaviours [22]. For example, a study by Hefner et al. showed an improvement in team learning and continuous improvement, an improvement in feedback and communication about errors and overall communication openness following a CRM training [23]. However, these initiatives are largely drawn on studies conducted in high-income settings, with very little emerging from resource-constrained low-and-middle-income settings, and particularly in the context of newborn care, yet the quality of maternal and newborn care are among the indicators of health system performance.

In this paper, we provide a detailed description of the nature of team processes in Kenyan NBUs, particularly, how this is enabled or restricted by intra- and interprofessional team dynamics. We also explore the contextual environments within which NBU teams operate and the implications of all these on the coordination practices in the delivery of newborn care. By providing this description, we highlight a set of core principles underlying team-based care, critical for safe delivery of newborn care, and provide a set of essential values that enable high-functioning teams. This, we hope will provide key learning on reducing the barriers to effective team-based neonatal care.

## Methods

### Study design

This exploratory study utilized an ethnographic approach, employing qualitative methods including non-participant observation, and in-depth interviews with health care providers to understand intra- and interprofessional teamwork and team processes in Kenyan newborn units.

### Setting characterization of Kenyan NBUs

In Kenya, newborn care is provided in both public and private facilities. In the public sector, newborn care is primarily provided at levels 4 and 5 (public county-level referral) hospitals (formerly, district hospitals), which are dynamic depending on the county context and in level 6 hospitals (national referral hospitals and large teaching private hospitals), which are highly specialized. Private sector facilities include faith-based mission hospitals, non-governmental (NGOs) and other privately owned facilities.

This study was therefore conducted in three purposively selected hospitals: one drawn from the private sector - a faith-based mission hospital and two level 5 public hospitals varying in county context - a rural and an urban county. Further, variations in the volume of newborn admissions were considered, ranging from a hospital with an average of thirty admissions to that with ninety admissions monthly. A summary of the study hospital structure and staffing is provided in Table 1 below.

Overall, while the structure and capacity of these hospitals vary, they all generally face major resource and performance challenges including high patient-to-staff ratios, high patient mortality, and inadequate bed capacity, more so in the public sector [24].

The high workloads and staffing shortages in these public NBUs [25] have been shown to lead to extensive informal task sharing/shifting, especially among nurses, nursing students, mothers, and support staff [26]. This has fostered emergent innovative and coping strategies among these cadres, with implications on who is accountable for patient care.

**Table 1:** A summary of hospital structure and staffing

Hospital	Description		
Hospital code	X	Y	Z
NBU bed capacity and no of admissions	7 incubators and 14cots with approximately 38 admissions monthly (456 yearly)	10incubators and 10cots with approximately 27 admissions monthly (324 yearly)	22 incubators and 48cots with approximately 90 admissions monthly (1080 yearly)
NBU staffing	15 Nurses 1 Medical officer 2 Clinical officers 1 Pediatrician	15 Nurses 0 Medical officers 2 Medical officer interns 4 Clinical officer interns 6 Clinical officers 3 Pediatricians	26 Nurses 5 Medical officers 4 Clinical officers 3 Pediatricians

### Study sample

The health care workers we interviewed were purposively selected by considering years of working experience in the unit, managerial position, and training level. In total, we conducted 17 interviews with twenty health care providers i.e., nine nurses, two nursing students (one group interview), three medical officer interns (one group interview), two clinical officers, one medical officer, two paediatricians and one neonatologist who were primarily involved in newborn care provision and had been observed during any of the shifts. Table 2 below provides a summary of the study sample.

**Table 2:** Study sample summary

Hospital	X	Y	Z	Total
In-depth interviews	Nurses – 2 2 Nursing students – (1interview) 3 Medical officer interns – (1 interview) Pediatrician – 1	Nurses – 3 Clinical officer – 1 Pediatrician 1	Nurses – 4 Clinical officer – 1 Medical officer – 1 Neonatologist – 1	17
Observation time of care process	85	82	83	250 h

### Data collection and analysis

To understand how the NBU teams are organized and how health workers interact within and across the cadres during care provision, the first author began by conducting non-participant observations of neonatal care provision processes across all three hospitals. The cadres observed included nursing students, nurses, clinical and medical officer interns, clinical officers, medical officers, and paediatricians/neonatologists. We conducted a total of 250 h of observations across the three study hospitals over nine months- from July 2018 to April 2019. These observations focused on understanding interactions within and between teams during care provision, particularly during handovers, ward rounds, and the patient admission process.

**Fig. 1:**
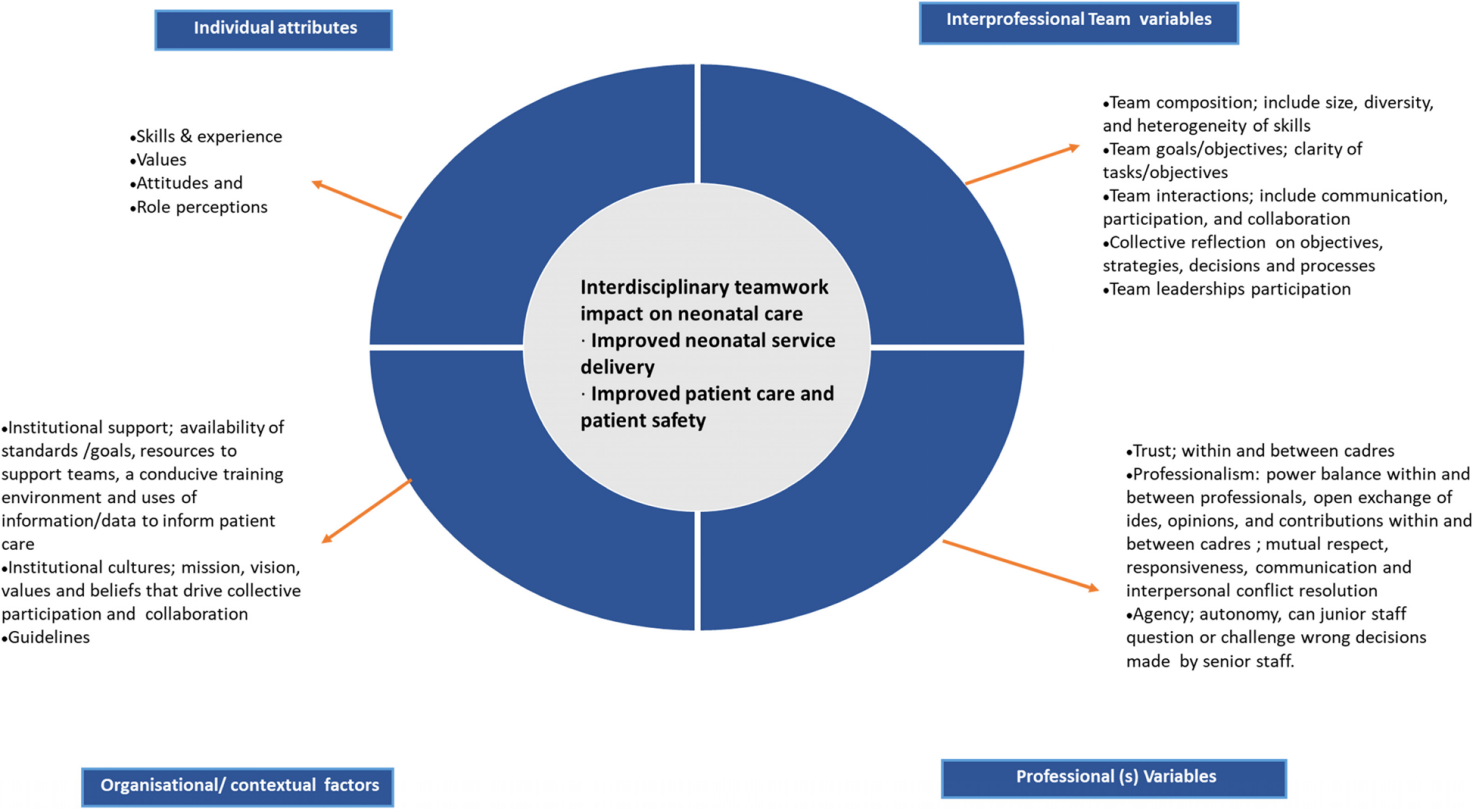
Conceptual framework on understanding intra- and interprofessional teamwork and team processes adapted from Cohen and Baileys (1997) Team Effectiveness Framework

To explore factors that may facilitate/undermine intra- and interprofessional teamwork, we adopted Cohen and Baileys’s Team Effectiveness Framework (Fig. 1) [27]. Within this framework, team effectiveness is conceptualized as a function of several factors including task, group, internal and external processes, as well as organizational factors [27, 28]. Key attributes and processes at the individual, team, organisational and contextual levels may influence teams’ behaviours and interactions as illustrated in Fig. 1 below. We drew on this framework, specifically on the concepts summarised in its quadrants, to explore teamwork in Kenyan hospitals’ NBUs.

We focused on the team and organizational/contextual variables to explore the ‘software elements’ of interprofessional teamwork. Our assertion is that team-level attributes such as composition and team goals/objectives provide insights into how teams are formed and what drives these teams towards achieving their desired goals. The (in) ability of teams to identify appropriate goals and demonstrate sustained commitment may further be influenced by professional variables: their ability to foster positive relationships, trust (existence of open trust between senior and junior staff and patients) and professionalism. More broadly, institutional support provides a favourable environment for collaborative teamwork as it ensures resources e.g. adequate staffing is available. Such favourable contexts support best practice initiatives and their implementation, facilitate rewards and benefits (motivation) for teams, and promote a positive interdisciplinary co-existence [29, 30].

Drawing on this framework, a semi-structured observation guide was developed, piloted, and consequently used to direct the observations as per the study objectives. Key aspects of teams explored in the observation guide included team composition, roles, leadership, and interactions such as communication mechanisms, etc. Detailed field notes were documented and insights, from observations and conceptual understanding from relevant literature, were used to develop an interview guide. The in-depth interviews with nurses and clinicians were semi-structured but were long-form [31] in nature which helped elicit narratives rather than “answers”.

The interviews were conducted in English, lasted 40-60 min, and were audio-recorded with consent from the study participants. All the audio files were then transcribed verbatim, the field notes typed, and interview transcripts were then imported to NVivo 12 for coding. A thematic content analysis approach [32] was adopted, which involved data familiarization, generating initial themes, grouping themes to broader conceptual themes by matching patterns, and relating these to existing literature. The first and the last author independently coded the data in the first phase of analysis. This was then followed by intensive discussions by the two authors and comparing emerging codes and developing a consensus on a final coding framework that mirrored the conceptual framework but also allowed emerging themes. The final coding framework was used to code the data and develop analysis charts in NVIVO 12. The coded data was drawn on to support categorization, inter-relationships, interpretation, and presentation of the final themes.

 In summary, all methods employed in this study were carried out in accordance with the relevant guidelines and regulations (i.e., as stipulated in the approved protocol).

## Results

We describe the nature of interprofessional teams working in NBUs of public hospitals under three main themes that include: (i) the dynamic nature of team composition; (ii) the role of team relationships in nurturing team climate and how these are influenced by iii) routines and rituals within institutions. Table 3 below provides a summary of the emerging themes, sub-themes, and illustrative evidence.

**Table 3:** A summary of emerging themes and sub-themes

Themes	Sub-themes	Key emerging findings; illustrative evidence provided under each theme below
1. Dynamic nature of team composition	‘Core’ or routine team	• Stable and permanent in constitution (members often physically present in NBU) • Minimal intra- and inter-cadre teamwork due to high patient to staff ratios • Consequently, care provision is largely fragmented, focused on task execution • Implicit goals/targets, that are assumed to be shared among providers • These targets are not just implicit but also ad hoc and reactional
	Emergency team	• Dynamic and time-related membership • Mostly constituted during scenarios/situations considered as emergency e.g., resuscitations • Often multidisciplinary, demonstrating efficient collaboration and coordination, and broken professional boundaries
	Temporary ad hoc teams	• Innovative informal teams assembled to cope with high human resource demands • Included participation of students, support staff and mothers in executing ‘core’ team tasks • Characterised by informal task-sharing and task-shifting • While it provided opportunities for patient-centered care, had negative consequences on the quality of care
2. The role of team relationships in nurturing team climate		• Positive relationships characterised by intra-cadre dependence and personal bonding appeared as a coping mechanism in this context • We observed lots of intra-cadre collaboration and support for one another, e.g. colleagues covered shifts/tasks for each other through local arrangements. • Interactions within the ‘core’ team encouraged learning, we observed lots of knowledge and skills sharing between nurses and junior doctors/clinical officers • Leadership practices adopted were key in promoting team cohesion and positive team working • E.g., Distributed forms of leadership promoted an open environment for interaction and collaboration rather than a hierarchical/authoritative one which undermined collective participation in decision-making. • Opportunities for information sharing and communication also promoted positive relationships: informal messaging apps such as WhatsApp emerged as useful spaces for both formal and informal communication
3. The value of routines and rituals to NBU teams		• Institutionalized formal and informal routines and rituals such as monthly departmental meetings, daily prayers, and morning devotions shaped team cohesion, by enabling bonding over shared values and beliefs • A mission driven institutional culture seemed to promote openness, and co-existence between teams • On the contrary, inefficient bureaucracy and inertia which seemed persistent in public sector limited opportunities for interactions in both formal and informal routines

### Dynamic nature of NBU team composition

In Kenyan newborn units, care is provided by a range of healthcare professionals that include nurses, nursing students, clinical officers [33]^1^, medical officers, clinical and medical officer interns, paediatricians/neonatologists, physiotherapists (in tertiary settings only), and nutritionists. Often, nurses and trainee clinicians are at the frontline, with support staff and mothers (who informally take on some tasks such as cleaning babies, due to staff shortages) being part of the interactions within these spaces.

Across the study hospitals, we observed that the size and accessibility of team members varied across shifts depending on the number of admissions and availability of staff. This ranged from a team of four to seven individuals (2–5 nurses, 1 clinical officer, and 1 medical officer) during the day shifts, to three to four individuals (3 nurses and a medical officer on call) during the night shifts, caring for thirty to eighty babies. Consequently, health care teams were often overstretched during work shifts with minimal opportunity for intra- and interprofessional interaction, as every individual within the team aimed at executing as many tasks as possible, therefore, limiting space for shared decision-making and overall collaboration in patient care.

Despite this organizational structuring, our observational data revealed that in practice, teams are conceptualized and operationalized differently. We observed variations in the day-to-day composition of healthcare teams in NBUs, with variations mainly linked to the physical and temporal stability of healthcare teams. Thereby, three types of work teams emerged in our findings, loosely categorized as the ‘core’/routine team, emergency team, and temporary ad-hoc team.

#### The ‘core’ or routine team

Across the study hospitals providers who were physically present in the unit for most of the day during their work shift were considered as the ‘core’ or routine team which was mostly stable and largely permanent in constitution. This team included nurses, student nurses, medical officers & clinical officer interns, who worked closely and often in consultation with senior colleagues who joined this core team during specific tasks such as ward rounds and events such as audit meetings.

*Mostly the nurses and the MO interns, we are very, very close with each other, but whenever it comes to a problem, the rest can come in though we are the very immediate care providers to the neonates, but the rest as well they are involved, and they come daily only that every time we are very close to the nurses and the MO interns with consultation from the other. (Medical Officer Intern)[Hospital X]*

Despite the perceived notion of a ‘core’ team, we observed that care provision within this team was fragmented, and largely driven by cadre-specific roles with minimal inter-cadre interaction, yet, these providers worked in the same space, providing care to the same patients. This could be explained by a lack of shared interprofessional goals, and where these were observed, they were often ad hoc and reactional. As such this team seemed to aim at executing shift to shifts tasks, with minimal opportunities for interprofessional learning.

*Personally, if I come to work, I interact with everybody, you do your work. I do my work. I give orders, you do this. Sometimes somebody is busy somewhere maybe they cannot do but you know there are different orders, clinical notes those must be done…you come you do your notes the nurse comes later fulfils the orders in the notes. (Clinical officer)[Hospital Z]*.

Furthermore, intra- and inter cadre teamworking was limited by the high patient to staff ratios. For example, where an average of four nurses cared for about eighty babies in a shift, they were often physically spaced out in various rooms limiting opportunities for intra-cadre teamwork. Similarly, there was an inconsistent presence of medical staff in NBUs, such as their absence during afternoon shifts which are perceived to be less busy [26].

#### The emergency teams

In addition to the ‘core’ or routine teams, which were often ‘stable’ across shifts, we observed dynamic time-related teams that we refer to as emergency teams, particularly in hospital Y. This teaming type emerged during emergency scenarios such as resuscitations, as demonstrated in the text below, we observed that in such scenarios, the ‘core’ team collaborated in patient care, while also escalating patient care to include senior colleagues such as medical/clinical officer on call and the consultant. Therefore, this team was often multidisciplinary, and characterised by efficient collaboration and coordination of care across professions, with silos’/boundaries being ‘broken’.

**Table:** 

*Thirty minutes into the night shift at about 1953hrs, all the three nurses covering this shift were at the nurse station discussing task allocation, same time a medical officer intern(MOI) rushed into the unit and went straight to the resuscitator. Earlier after handover, the nurses had set this resuscitator in anticipation to receive a preterm baby, the MOI picked something and headed back to the door. Nurse B quickly told him, “I have seen you the MOI responded, “I have been sent” nurse B then said, “you are not meant to pick anything from that resuscitator, but just go …”. About two minutes later, a clinical officer intern (COI) rushed in with a baby, and placed the baby on the resuscitator, immediately, the MOI who had just left the unit came in. All the three nurses quickly rushed to the resuscitator, nurse B started bagging the baby, while nurse A was putting on the monitor. The qualified clinical officer (CO) on call came in running, she rushed to the resuscitator, and took over the resuscitation while giving instructions to the nurses on what to get/ drugs to prepare for administration. The present team was now crowded at the resuscitator each one trying to help with the resuscitation…the MOI then immediately called the consultant and informed him of this baby and what they were doing. The consultant on call came in 2 minutes later, there were now a team of 8 people all standing by the resuscitator, trying to help where they could. (JJ Field notes)[Hospital Y]*	

With the silos broken, we observed more team cohesion and a shared drive for patient care and therefore more patient-centred care and more learning occurred, as demonstrated by initiatives taken by emergency teams in hospital Y, such as audits conducted within twenty hours of infant mortality, to primarily reflect on provided care and address any gaps in care [34].

#### Temporary ad-hoc teams

Beyond the ‘core’ and emergency teams that solely involved healthcare providers, we observed the emergence of innovative, informal teams often quickly assembled to cope with high demands in the NBU. This was especially the case for hospitals X and Y. These teams were primarily characterised by the participation of the ‘core’ team, unsupervised students, support staff and mothers in varying constitutions depending on the level of tasks and staff availability in the NBU. The formation and organization of this team type was often driven by extremely low staff to patient ratios and competing demands that often made working in the NBU an impossible job [32]. In this context, tasks such as administration of fluids were delegated to nursing students (at various levels of training with minimal supervision) and to mothers who were involved in the feeding of babies via nasogastric tubes and top tailing, encouraging organic task shifting to accomplish tasks officially expected of the NBU ‘core team’ but impossible to achieve with available staff ratios. We noted that although the delegation of tasks allowed the continued provision of care, the care provided was sometimes rationed, and erroneous and because there was limited cohesion among members of these temporary teams, very little learning occurred.

**Table:** 

*In today’s morning shift, there were two nurses (the nurse manager and his deputy are away in a seminar), two doctors and about twenty-one students, caring for seventy-six admissions (total newborns in the unit today was greater than 100% bed occupancy, therefore, neonates share cots/incubators). One nurse was filling in as the ward manager, she was expected to do managerial duties and her duties for the day, while the other nurse was tasked to administer medication to the babies. Given the high number of students in the unit, every room had at least 3 students. The nurses seemed to have so much to do but with very little capacity, as a result, they had delegated some of the nursing tasks: the students were now administering fluids, implementing suggestions from ward rounds and taking vitals with very minimal supervision. The nurse would step in only if they came to ask something they were not sure about.* *At feeding time, mothers came into the unit, those who were either cup feeding or feeding via nasogastric tube made their way to collect cups as they prepared to feed their babies, one mother didn’t seem sure of the amounts of feed/milk to express for their baby, given that the nurses were not present in this room, she went ahead to ask a nursing student, who checked the moms’ file to ascertain the prescribed feeds. Overall, the students seem to do a lot including supporting and supervising mothers during feeding with minimal supervision from the qualified nurse. (JJ Field notes)[Hospital Z]*	

Furthermore, while the delegation of tasks to mothers would have created an opportunity for more involvement in the care of their babies, we noted that they were only used as ‘extra hands’ to unbundle tasks and lacked more meaningful engagement. 

### The role of team relationships in nurturing team climate

As teams formed depending on prevailing ward contexts, relationships particularly intra- and inter cadre team relations continued to be shaped by the high patient to staff ratios, leading to emerging positive relationships, including intra-cadre dependence and personalized bonding as a way of coping. Consequently, the reliance on local norms, often a form of street-level bureaucracy to avoid what was perceived as onerous unsupportive processes emerged. For example, within the nursing and doctors’ teams, staff often made ‘local arrangements’ to cover shifts for each other, deviating from the official duty rota, without informing their managers.

*In terms of working, if something comes up as an emergency and am not able to cover my duty my team has always been available to cover me without having to take time off, so I would come and pay them back duties…so it is a good team we help each other whenever we can both at work and outside… so when a colleague asks you to cover for them because they are unwell and they wouldn’t want to take a sick off because that will mean that person doing that duty as an extra duty on top of what they are doing, so we would just ask our colleagues to cover for us…(Medical officer)[Hospital Z]*.

In addition, between cadres, positives relationships emerged from the existing team types, which seemed to shape how care is provided and determined opportunities for interprofessional team learning. For example, we observed lots of knowledge and skills sharing between the ‘core’ team i.e., nurses and trainee clinicians/junior doctors. For instance, where trainee clinicians were uncertain or unclear about certain procedures such as calculating drug dosages, nurses were their first point of contact to seek guidance and assistance.

*Yes there are some things that to be honest I believe that the nurses would know more than I do like for example when I came here I had not used something like a CPAP machine and the nurses had been trained on it some time so when I would put the baby on the CPAP they would tell me that in accordance to their experience a baby with such a condition will not do well on CPAP or if I could just put a baby on oxygen now they can say we have a CPAP machine and now we can try, you know if I give this kind of medication sometimes maybe they will say a baby with this kind of you know sickness for this long don’t you think we need to give thi*s...(*Medical Officer Intern*)[*Hospital X*]

Overall cohesion within interprofessional teams appeared to be further influenced by leadership styles adopted by the team leads, e.g., we observed how facilitative, shared and distributed leadership (i.e., where leadership is shared among team members rather than focused on a single designated leader, therefore, leadership roles are performed by multiple members of the team) promoted an open environment for interaction and collaboration. For example, in hospital X we observed delegated autonomy by the nurse manager to the nursing team, in the day-to-day running of the unit, conferring not only autonomy but enthusiasm to frontline line nurses who were inspired to lead the execution of administrative tasks such as the ordering of supplies, and overall management of the unit. In such an environment, we further observed that nursing team members would openly raise suggestions and concerns without any fear of intimidation and were empowered to participate in shared decision making, coordination of tasks, and building trust.

*I think the pediatric department here we are very much, it is not a ‘do what I say or it is not a hierarchy as much as we need a coordinator so when there are big questions it is easier to have a point person to go to, so XX and I, I mean we work together if she sees a problem at the nursery she would call me but then all the nursing managers know and clinical officers (Cos), come to me directly but then the nursing managers, the coverage nurses, we generally call XX with the problem and then she will disseminate the information back down and she usually uses a phone call not texting to communicate that information. (Pediatrician) [Hospital Y]*.

Positive relationships and cohesion within cadres were further reinforced by opportunities for information sharing and overall communication. For example, informal messaging applications such as WhatsApp not only increased social comradery but also emerged as safe spaces for official business e.g., sharing duty rosters, and communicating planned meetings. Doctors often used WhatsApp to seek technical advice from colleagues and seniors who were off duty and for the handover of critical patients. For nurses, missed care or any mistakes that had occurred were discussed and corrected on this platform. The unique value of these platforms was that they enabled a general non-personalized approach which was often taken to avoid the victimization of team members and to foster team cohesion.

*…Communication gets across everyone in that group, so it is very easy, and most people are online, so it is very easy to get these messages, for example, schedules are posted in that group… so all that has been a good aspect of it …issues are also sorted out there where need be if there is someone not happy about an event an occurrence then it is still aired there and people troubleshoot if there is a difficult case for example in the night when you are on call or something you can still post it there people give the opinions on a particular area so that is a very nice way of communicating. (Clinical officer)[Hospital Y]*.

### The instrumental value of routines and rituals to NBU team﻿s

The value of software elements in shaping NBU teams was seen in how cultures emerged, how values were strongly held and how day-to-day routines and rituals within institutions shaped team cohesion and overall functioning of teams. For example, in hospital Y, ‘informal’ routines and rituals such as morning devotions, praying together before morning change of shift handovers, and before ward rounds reinforced a sense of fellowship and togetherness between team members. Consequently, bonding over shared values (i.e., Christian values), mutual respect, and an unspoken but shared sense of working towards a ‘higher good’ promoted cohesion within interprofessional team members.

**Table:** 

*This morning I joined the nursing team (night and day shift teams: 12 nurses and 8 nursing students) who had already begun their morning devotion meeting, two nurses coordinated the bible reading, singing of hymns, and prayers. Immediately after the prayer session, one nurse who I later learnt had been selected to join a committee on promoting culture change in the hospital made an oral presentation, she talked to the team about the hospital values which included compassion, accountability, employee engagement, sustainability, professionalism, and efficiency. She discussed each, presenting on what behaviours show the values, what is expected of employees and what is not expected of them. After the presentation, she asked the team if they had any comments, the NBU in charge said, “Attitude matters a lot, we need to have a positive attitude towards our work…for you as a nurse, you need to have a sixth sense…remember we are a Christian institution…take initiative, call in the doctor when you observe something that needs their attention, don’t assume and feel or say it’s not your work. When a lab results as come in, check if you have any concern, inform the doctor…”(JJ Field notes)[Hospital Y]*

*…We are also a Christian institution…so it is just in the good nature of Christianity that we do not create rifts but we live harmoniously with each other and solve issues that you face openly without hiding them so and as the slogan goes it is health that God’s glory, so it is not your work you are working towards, it is the glory of God at patient care. (Clinical officer) [Hospital Y]*.

Of note was how hospital Y ‘s missionary-driven culture promoted openness, and co-existence within and between cadres, but was also leveraged as a strategy to move teams towards a collective strive of executing team tasks and activities in a unified manner to provide optimal care. So while team goals were not officially articulated, these informal routines and values were certainly a useful avenue for getting people to work together.

*I think openness which means that there is no high-handedness where someone feels like since am at this position then you are nothing, I mean you cannot do anything, you cannot talk to me you have to go through these channels to reach me with that being open and leading by example that means that people easily interact; I think that over time that is what has been enforced. (Clinical officer) [Hospital Y]*.

While drawing on the mission and values of hospital Y provided agency for a continued strive to provide the best care to the patient among providers, inefficient bureaucracy and inertia seemed to be persistent in the public sector, where staff experienced apathy towards improvement with notably fewer opportunities for further formal interactions lacking. For instance, while there were some attempts at organizing meetings aimed at bringing all cadres together, individuals generally lacked interest. They cited monotony due to extensive meetings discussing similar issues over and over without any action or positive outcomes.

*That is what I am saying we go (to departmental/audit meetings) to discuss the same thing over and over again so it is monotonous this is government things are monotonous we go there we are discussing this the meeting takes three hours we are just going around and around. (Clinical officer) [Hospital Z*.

Nonetheless, across all hospitals, we observed informal spaces that provided opportunities for communication and engagement within teams in the NBU e.g., tea-break sessions, mainly in hospitals X and Z were an important part of the nurses’ shift to stop, reflect, share, vent and encourage each other to get through the rest of the shift. Interestingly, it was only the nursing team that bonded over tea breaks, with only a few instances where the clinical and medical officers would join in perhaps because the latter were not physically present in the NBU for long. This suggests that like the utilization of both formal and informal routines and rituals for interprofessional interactions was sub-optimal.

## Discussion

Although research has shown the value of interprofessional teamwork and collaboration in healthcare, existing studies have mainly focused on teamwork and team processes in high-income settings and have not examined how teams function in resource-constrained low- and middle-income settings and more particularly in the context of newborn care. Our work contributes to existing literature that highlights how context shapes the day-to-day work arrangements, by highlighting team formation dynamics and interaction processes that are largely influenced by institutional and contextual factors.

The structural characteristics of teamwork are theorized to be shaped by institutional, contextual and relational factors including relationships between healthcare providers and patients [35]. In our findings, we have provided empirical evidence to support this assertion. We have demonstrated how the emergence of various team types was linked to the prevailing contextual factors i.e., the high patient to staff ratios as demonstrated by Murphy and colleagues’ study which indicates a nurse: baby ratio of 1:15 in Nairobi’s public hospitals [32]. These team types were characterised by features linked to the physical and temporal stability of health care teams, for instance, the ‘core’ team was considered more stable and mostly permanent compared to emergency teams that are assembled to address certain situations within the newborn care units. On the other hand, expertise and membership within the ‘emergency’ team were diverse with more formal leadership present (i.e., in this context attending paediatricians/neonatologists) [3]. The unique contribution of ‘temporary ad-hoc teams composed of students and mothers in care, is that they contributed to the informal practice of organic task-shifting as a coping response to overwhelming workloads and staff shortages, but, with implications on the quality of care delivered e.g., missed and rationed care [36, 37]. Additionally, the context not only influenced team typology, but also the ability of these teams to identify common goals and to foster collective working and commitment towards achieving unified aims. Ideally, these goals ought to be centred on the nature of its institutional mission and vision statements, under which shared values can be drawn to motivate interprofessional team performance [38, 39].

Within the different team types, the intra- and interprofessional relationships amongst team members were further influenced by individual and group level contextual factors. In this study, we observed how positive familial interactions composed of collegial and emotional support among the ‘core’ team members (i.e., between nurses and junior doctors/trainees), supported coping with day-to-day work pressures in comparison to nurses and senior doctors whose relationships were more distant. Furthermore, the nature of relationships among care teams had implications on patient care. For example, professional hierarchies limit effective and timely communication of patient information between care providers, leading to delays in patient care and consequently persistence of professional’s silos [40].

Core to the development of functioning teams and nurturing team cohesion was the development and strengthening of intra- and interprofessional team leadership practices [41, 42]. The value of distributed leadership (i.e., where leadership is shared among team members rather than focused on a single designated leader, therefore, leadership roles are performed by multiple members of the team) in promoting team effectiveness was highlighted [43]. While our findings suggest how the existence of such a leadership approach in one of our study sites promoted shared decision making, coordination of tasks, and building trust, research on leadership in Kenyan healthcare settings reports a lack of effective training for managers/team leaders in clinical settings on crucial leadership skills [44]. As such, managers/team leaders are often unprepared to execute their roles and rely on their ideas, professional expertise and personal values to undertake these roles [45]. As a result, profession-specific team leads emerge, (i.e., nurse managers for the nurses and medical consultants for the doctors), undermining distributed leadership practices that would promote collective decision-making practices among team players, and across professions.

Additionally, NBU leadership practices were driven by institutional context: cultures and norms within institutions [44]. For example, a missionary-driven culture promoted openness, co-existence within teams and collective striving towards achieving ’higher good’, therefore acting as a supportive environment for fostering good leadership practices. In the absence of positive cultures, good leadership practices may be undermined. Therefore, initiatives such as training and coaching of leaders ought to recognize the cognitive and behavioural capacities (e.g., training to improve communication and emotional competence) required for facilitative leadership, alongside technical leadership skills) [46, 47].

The value of relational elements in leading, coping with stressful situations and ensuring shared commitment has been shown [6, 47]. Further, studies suggest that certain institutionalized routines and rituals can help direct day-to-day performance and interactions of professionals performing clinical tasks and can facilitate or undermine teamwork [48]. These routines and rituals also provide opportunities for leaders/managers to reinforce institutional values and beliefs and guide team behaviour and performance [49]. In our context, we observed how health workers used prayer meetings and tea breaks as opportunities for seeking a sense of togetherness and consequently ‘unspoken goals’ therefore promoting interprofessional team working. These further suggest that while routines and rituals provide opportunities for interprofessional interactions, they are often not optimally tapped on as a collective resource for advancing intra- and interprofessional interactions and teamwork.

In summary, we have demonstrated the influence of contextual factors on team structures and the day-to-day functioning of health care teams and that routines and rituals shape day-to-day interactions among professionals. Teamwork is influenced by relational and broader contextual factors. All these factors are crucial when designing behaviour change interventions aimed at improving interprofessional team working in practice.

## Conclusions

 Our findings suggest that team structures/composition are largely influenced by the prevailing contextual dynamics, as such, health care teams in NBUs vary depending on time and prevailing situations. We also highlight how the emergence of various team types shapes the nature of relationships between care providers. Local leadership practices are key in promoting positive team relationships and intra- and interprofessional teamwork, such skills must be actively developed through training and coaching leaders to strengthen their cognitive and behavioural capacities.

Overall, these interventions may need to be tested and evaluated to ensure that they are context-appropriate. As demonstrated, the role of context, i.e., variability in staffing complements and institutionalized routines and rituals cannot be ignored; therefore, when thinking about interventions to promote teamwork at all levels of care, strategies may also be needed to develop and reinforce shared goals and values amongst all staff.

## Supplementary Information

**Additional file 1.**

**Additional file 2.**

## Data Availability

To maintain the anonymity of the study participants (given a small sample thus easily identifiable), the datasets used and/ or analyzed during this study are available from the authors upon reasonable request and with permission of the KEMRI-Wellcome Trust Research and Governance committee.

## Footnotes

1 Clinical officers (COs) are non-physician clinicians, they are mid-level healthcare providers who have received less training and have a more restricted scope of practice than physicians. In Kenya, they are regulated by the Clinical Officers Council, who oversee their training, registration and licensing.

## Declarations

Further, written informed consent was obtained from all study participants. The ethics review committee approved the use of both written (for the interviews) and verbal (for the observations) informed consent in this study. Written informed consent was sought from all study participants before the interviews were conducted. For the observation of care provision processes, at the beginning of every shift, a brief introduction of the study and the role of the researcher in the unit were highlighted, following which verbal consent was sought from all providers present. This was done across the various shifts: morning/afternoon/night or day/night across the study hospitals were observed. This approach allowed for a continued consent process, where participants had an opportunity to express discomfort (if any) with being observed. We documented verbal consent as part of the field notes.
