# Supplementary material for: Understanding intra- and interprofessional team and teamwork processes by exploring facility-based neonatal care in kenyan hospitals
Source: BMC Health Serv Res. 2022 May 13;22:636. doi: 10.1186/s12913-022-08039-6 (PMC9103056; doi:10.1186/s12913-022-08039-6)
Supplement: Supplementary file 1 — Additional file 1. [file 12913_2022_8039_MOESM1_ESM.docx]

**Non-participant observation guide for neonatal care provision processes**

1. Team composition

- How do teams providing neonatal care look like? (cadre differences, hierarchy level within the cadres, gender differences)
- What are the roles of each team member? How is it allocated? What are the forms of interactions in reference to team characteristics?
  - Are there procedures for communication? Is it Adhoc? Open channel of communication? Planned/scheduled e.g. debriefing, handover etc.
  - Are there documents or policies that describe roles and responsibilities, especially as there are so many “trainees” nursing students, MO interns, CO interns, Family Medicine MMed students, visiting external trainees, etc.

1. Leadership

- Observe managers (? who are you labeling a manager? the consultant? The shift lead? the official nursery manager-ie Bishop?) of the various professional teams in the NBU
- How do they communicate with staff?
- Do they encourage interactions among staff and other cadres?
- Does the leadership provide an environment for support of members to freely discuss issues relating to quality and patient safety?

1. Team interactions

- Observe how information is shared
- Verbal, leaflets, WhatsApp, time info is shared and by who to whom, frequency, nature of information and when (are you aware of the WHATSAPP groups that the pediatricians/obstetricians use at Kijabe?)
- How do teams interact within and between cadre? Mode of communication? Gauge the tone and body language when communicating? (This body language implies face to face-but you may need to consider tracking WHATSAPP communication-at least at Kijabe where it is used a lot)
- Interactions within cadres; how do junior and senior staff interact?

1. Trust

- Do all team members express their thoughts and opinions without fear? (note who is saying what and responding to whom (Junior vs senior staff, within senior staff and within junior staff). (How do you describe fear? )
- How are they communicating/discussing these issues -gauge the tone, facial expressions, pauses etc)?

1. Professionalism

- How are ideas exchanged between and within different professional cadres? Observe how difference of opinion is being resolved

1. Agency

- Observe if there are differential power issues between and within staff cadres?

1. Context

- Observe resources available for use by health care teams; space, basic materials such as notebooks, pens, tea?
- How often do hospital managers visit the NBU? If they do, how do the NBU staff engage the hospital administration? e.g. when asking for supplies? More staff?
